# Preference of Conjugated Bile Acids over Unconjugated Bile Acids as Substrates for OATP1B1 and OATP1B3

**DOI:** 10.1371/journal.pone.0169719

**Published:** 2017-01-06

**Authors:** Takahiro Suga, Hiroaki Yamaguchi, Toshihiro Sato, Masamitsu Maekawa, Junichi Goto, Nariyasu Mano

**Affiliations:** 1 Graduate School of Pharmaceutical Sciences, Tohoku University, 1–1 Seiryo-machi, Aoba-ku, Sendai, Japan; 2 Department of Pharmaceutical Sciences, Tohoku University Hospital, 1–1 Seiryo-machi, Aoba-ku, Sendai, Japan; University of Parma, ITALY

## Abstract

Bile acids, the metabolites of cholesterol, are signaling molecules that play critical role in many physiological functions. They undergo enterohepatic circulation through various transporters expressed in intestine and liver. Human organic anion-transporting polypeptides (OATP) 1B1 and OATP1B3 contribute to hepatic uptake of bile acids such as taurocholic acid. However, the transport properties of individual bile acids are not well understood. Therefore, we selected HEK293 cells overexpressing OATP1B1 and OATP1B3 to evaluate the transport of five major human bile acids (cholic acid, chenodeoxycholic acid, deoxycholic acid, ursodeoxycholic acid, lithocholic acid) together withtheir glycine and taurine conjugates via OATP1B1 and OATP1B3. The bile acids were quantified by liquid chromatography-tandem mass spectrometry. The present study revealed that cholic acid, chenodeoxyxcholic acid, and deoxycholic acid were transported by OATP1B1 and OATP1B3, while ursodeoxycholic acid and lithocholic acid were not significantly transported by OATPs. However, all the conjugated bile acids were taken up rapidly by OATP1B1 and OATP1B3. Kinetic analyses revealed the involvement of saturable OATP1B1- and OATP1B3-mediated transport of bile acids. The apparent *K*_m_ values for OATP1B1 and OATP1B3 of the conjugated bile acids were similar (0.74–14.7 μM for OATP1B1 and 0.47–15.3 μM for OATP1B3). They exhibited higher affinity than cholic acid (47.1 μM for OATP1B1 and 42.2 μM for OATP1B3). Our results suggest that conjugated bile acids (glycine and taurine) are preferred to unconjugated bile acids as substrates for OATP1B1 and OATP1B3.

## Introduction

Bile acids are amphipathic steroidal molecules derived from cholesterol catabolism. They are conjugated with amino acids, glycine and taurine, before being secreted into the bile. Cholic acid (CA) and chenodeoxycholic acid (CDCA) are the primary bile acids in humans. Secondary bile acids, deoxycholic acid (DCA) and lithocholic acid (LCA), are produced by gut microbiota from the primary bile acids. Bile acids play essential roles in many physiological functions such as digestion and absorption of lipids and lipid-soluble nutrients in the intestinal lumen, protection against bacterial overgrowth, and elimination of cholesterol from the body [1]. As signaling molecules, bile acids are involved in the regulation of glucose metabolism [2], energy expenditure [3], and cellular immunity [4, 5]. Individual bile acids display different potency in activating their nuclear receptors, including farnesoid X receptor (FXR) [6–8], pregnane X receptor [9], and vitamin D receptor [10], and cell surface receptors, including G protein-coupled receptor TGR5 [4, 11], sphingosine-1-phosphate receptor 2 (S1PR2) [12], and muscarinic receptors [13]. FXR and TGR5 are strongly activated by CDCA and secondary bile acids, respectively, while both S1PR2 and muscarinic receptors are activated by conjugated bile acids [14]. Therefore, kinetics data for individual bile acids may be a useful tool in understanding the physiological functions.

Bile acids are synthesized from cholesterol in the liver, excreted into the intestinal lumen via the bile duct, reabsorbed in the ileum, and returned to the liver, thus completing the enterohepatic circulation [15–17]. Hepatic uptake, the last step in enterohepatic circulation, is the key process in regulating the amount of bile acids in peripheral as well as enterohepatic circulation. The hepatic uptake of bile acids involves Na^+^-dependent transport by Na^+^-taurocholic acid cotransporting polypeptide (NTCP) and Na^+^-independent transport by organic anion-transporting polypeptides (OATPs) [18]. OATP1B1 and OATP1B3 are members of liver-specific subfamily of OATPs, which contribute to the hepatic uptake of a wide variety of endogenous and exogenous compounds [19, 20]. Previous studies reveal that OATP1B1 and OATP1B3 transport several bile acids such as CA [21], taurocholic acid (TCA) [21–26], glycoursodeoxycholic acid (GUDCA), and tauroursodeoxycholic acid (TUDCA) [27]. However, comprehensive analysis of OATP1B1- and OATP1B3-mediated transport of bile acids has not been performed yet. Therefore, the transport properties of five bile acids (CA, CDCA, DCA, ursodeoxycholic acid, LCA) together with their glycine and taurine conjugates mediated by OATP1B1 and OATP1B3 were investigated using OATP1B1- and OATP1B3-overexpressing HEK293 cells. Our results indicate that glycine and taurine conjugated bile acids are preferred to unconjugated bile acids as substrates for OATP1B1 and OATP1B3.

## Materials and Methods

### Chemicals

**Fig 1:**
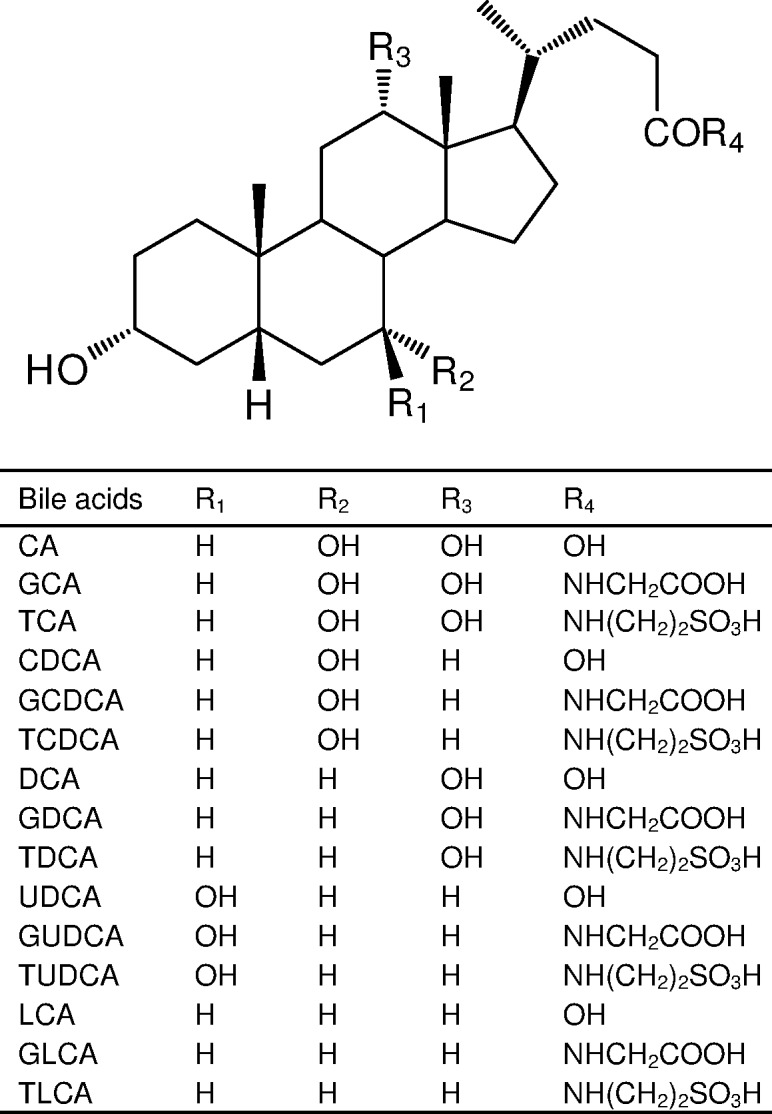
Chemical structures of bile acids.

CA, CDCA, and taurolithocholic acid (TLCA) were purchased from Sigma−Aldrich (St. Louis, MO). DCA and LCA were purchased from Wako Pure Chemical Industries (Osaka, Japan). TCA, ursodeoxycholic acid (UDCA), GUDCA, and TUDCA were purchased from Nacalai Tesque, Inc. (Kyoto, Japan). Glycocholic acid (GCA), glycochenodeoxycholic acid (GCDCA), glycodeoxycholic acid (GDCA), glycolithocholic acid (GLCA), taurochenodeoxycholic acid (TCDCA), and taurodeoxycholic acid (TDCA) were synthesized in our laboratory using previously reported method [28]. Internal standards (3, 7, 12-[^18^O]CA; 3, 7-[^18^O]GCA; 3, 7-[^18^O, ^2^H_2_]GCDCA; 3, 12-[^18^O, ^2^H_2_]TDCA; 3-[^18^O, ^2^H_2_]GLCA; 3-[^18^O, ^2^H_2_]TLCA) used for bile acids determination, were synthesized in our laboratory. Chemical structures of bile acids are shown in Fig 1. Ultrapure water was prepared using a PURELAB ultra apparatus (Organo Co., Ltd., Tokyo, Japan). All other chemicals were analytical grade and solvents were HPLC or LC/MS grade.

### Cell culture

Human embryonic kidney (HEK293) cells transfected with OATP1B1, OATP1B3, or an empty vector were previously established [29, 30]. OATP1B1/HEK293, OATP1B3/HEK293 cells, and mock cells were grown in DMEM supplemented with 10% fetal bovine serum and G418 (0.5 mg/mL) at 37°C under 5% CO_2_ and 95% air.

### Preparation of stock solution and calibration standards

Stock solutions of each bile acid were prepared in DMSO at a concentration of 40 mM. IS solution (100 μg/mL each) was prepared in ethanol/water (50:50, v/v). All stock solutions were stored at 4°C. Calibration standards were prepared from stock solution in methanol/water (50:50, v/v) at concentrations of 1, 2, 5, 10, 20, 50, and 100 nM.

### Transport study

Cellular uptake of bile acids was measured using monolayer cultures grown on poly-L-lysine coated 24-well plates. Cells were seeded at density of 2.0 × 10^5^ cells/well with G418-free culture medium for two days. Cell culture medium was replaced with fresh G418-free culture medium 24 h before transport assay. To examine bile acid transport, cells were washed once and preincubated with Krebs-Henseleit (KH) buffer (118 mM NaCl, 23.8 mM NaHCO_3_, 4.83 mM KCl, 0.96 mM KH_2_PO_4_, 1.20 mM MgSO_4_, 12.5 mM HEPES, 5.0 mM D-glucose, and 1.53 mM CaCl_2_, adjusted to pH 7.4) at 37°C for 10 min. Uptake was initiated by adding KH buffer containing each bile acid. At designated time, uptake was terminated by removal of incubation buffer and addition of ice-cold KH buffer. Cells were washed twice with ice-cold KH buffer. All bile acids were dissolved in DMSO with final concentration of DMSO less than 0.5%. The protein content of the solubilized cells was determined by Bradford method using a Protein Assay kit (Bio-Rad Laboratories Inc., Hercules, CA) with bovine serum albumin used as standard. The kinetic parameters, *K*_m_ and *V*_max_ were calculated by fitting the data of uptake rate of bile acids to Michaelis-Menten equation.

### Sample preparation

Bile acids taken up into cells were measured by using liquid chromatography/tandem mass spectrometry (LC/MS/MS). After terminating bile acid uptake, the cells were scraped and homogenized in 200 μL of methanol/water (50:50, v/v) containing internal standard, and transferred into a 1.5 mL polypropylene tube. Cell lysates were deproteinized by adding equal volumes of acetonitrile. The mixture was vortexed vigorously for 10 s and centrifuged at 15,000 × *g* for 5 min at 4°C. The supernatant was transferred into another 1.5 mL polypropylene tube and evaporated at 40°C. The residue was reconstituted in 50 μL of methanol/water (50:50, v/v), and 10 μL aliquots were injected into the LC/MS/MS system.

### LC/MS/MS conditions

Chromatographic separation of bile acids was carried out using Agilent 1290 System. Aliquots of samples (10 μL) were injected by auto-sampler and the analytes were separated on Inertsil ODS-3 (10 mm × 1.5 mm I.D., 5 μm) as a guard column and Inertsil ODS-3 (150 mm × 2.1 mm I.D., 5 μm) as an analytical column. The mobile phase consisted of 20 mM ammonium acetate (pH 6.8)/methanol (25:75, v/v) with a flow rate of 0.3 mL/min. The column temperature was maintained at 40°C. Analysis was carried out using an Agilent 6460 LC/MS/MS system. Negative ion electrospray parameters were set as follows: gas temperature, 350°C; gas flow, 12 L/min; nebulizer gas, 60 psi; sheath gas temperature, 400°C; sheath gas flow, 12 L/min; capillary voltage, 5000 V; and nozzle voltage, 500 V. Selected reaction monitoring (SRM) was performed for monitoring the transition, and SRM parameters are summarized in S1 Table. Data were collected and processed using MassHunter workstation software (version B.06.00) (Agilent Technologies, Inc.).

### Statistical analyses

Data are expressed as mean ± S.E (n = 3). When appropriate, the differences between groups were tested for significance using unpaired Student’s *t*-test. Statistical significance was defined as *p*<0.05.

## Results

### Time-dependent uptake of bile acids by OATP1B1 and OATP1B3

**Fig 2:**
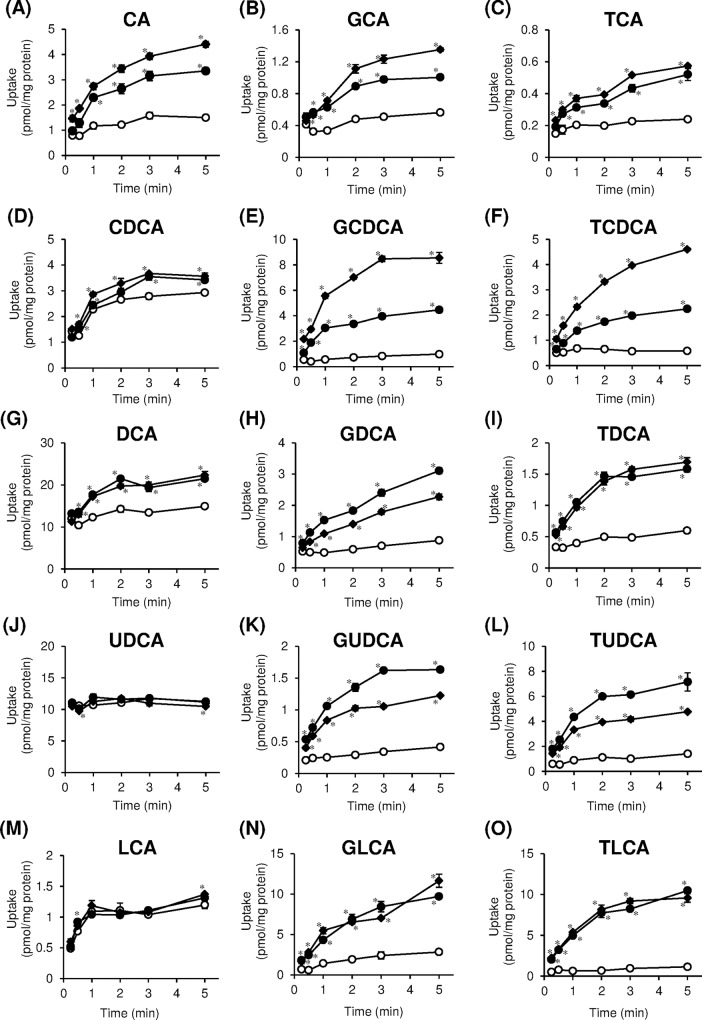
Time-dependent uptake of bile acids by organic anion-transporting polypeptides (OATP) 1B1 and OATP1B3. OATP1B1-overexpressing (closed circles), OATP1B3-overexpressing (closed diamonds), and vector-transfected (open circles) HEK293 cells were incubated for indicated times at 37°C. (A) CA (5 μM), (B) GCA (2.5 μM), (C) TCA (1 μM), (D) CDCA (0.1 μM), (E) GCDCA (1 μM), (F) TCDCA (0.5 μM), (G) DCA (1 μM), (H) GDCA (1 μM), (I) TDCA (1 μM), (J) UDCA (1 μM), (K) GUDCA (1 μM), (L) TUDCA (2.5 μM), (M) LCA (0.01 μM), (N) GLCA (0.2 μM), (O) TLCA (0.2 μM). Each point represents the mean±S.E. (n = 3). *p < 0.05, significantly different from vector-transfected cells by Student’s *t*-test.

We examined the uptake of CA, CDCA, DCA, UDCA, LCA, and their corresponding glycine and taurine conjugates by OATP1B1 and OATP1B3 in OATP1B1- and OATP1B3-overexpressing HEK293 cells, respectively. CA, the unconjugated bile acid, was taken up by OATP1B1/HEK293 and OATP1B3/HEK293 cells more rapidly than mock cells (Fig 2(A)). OATP1B1- and OATP1B3-mediated uptake of CA reached a steady state at 5 min. At this incubation time point, the uptake of bile acids by OATP1B1/HEK293 and OATP1B3/HEK293 cells was 2.2 and 2.9 times higher, respectively, than that of mock cells. Significant increase in time-dependent uptake of CDCA and DCA mediated by OATP1B1 and OATP1B3, respectively, was observed; however, the increase in uptake was less than 1.5 times in comparison with mock cells (Fig 2(D) and 2(G)). The time-dependent increase in uptake of LCA was observed in mock cells; however, OATP1B1/HEK293 and OATP1B3/HEK293 cells did not demonstrate enhanced uptake of LCA. (Fig 2(M)). No significant increase in the uptake of UDCA was observed in OATP1B1/HEK293 and OATP1B3/HEK293 cells in comparison with mock cells (Fig 2(J)). The conjugated bile acids were taken up by OATP1B1/HEK293 and OATP1B3/HEK293 cells more rapidly than mock cells (Fig 2(B), 2(C), 2(E), 2(F), 2(H), 2(I), 2(K), 2(L), 2(N) and 2(O)). OATP1B1- and OATP1B3-mediated uptake of most of conjugated bile acids reached a steady state at 5 min. At 5 min incubation time point, the uptake of bile acids by OATP1B1/HEK293 and OATP1B3/HEK293 cells were 1.8–9.2 times and 2.4–8.7 times higher, respectively, than those of mock cells. OATP1B1- and OATP1B3-mediated uptake of CA, GCA, and TCA increased linearly up to 0.5 min, and those of the GCDCA, TCDCA, GDCA, TDCA, GUDCA, TUDCA, GLCA, and TLCA increased linearly up to 1 min. Therefore, kinetic analyses of CA, GCA, and TCA were carried out at 0.5 min, and those of the other bile acids were carried out at 1 min.

### Kinetic analysis of bile acid transport by OATP1B1 and OATP1B3

**Fig 3:**
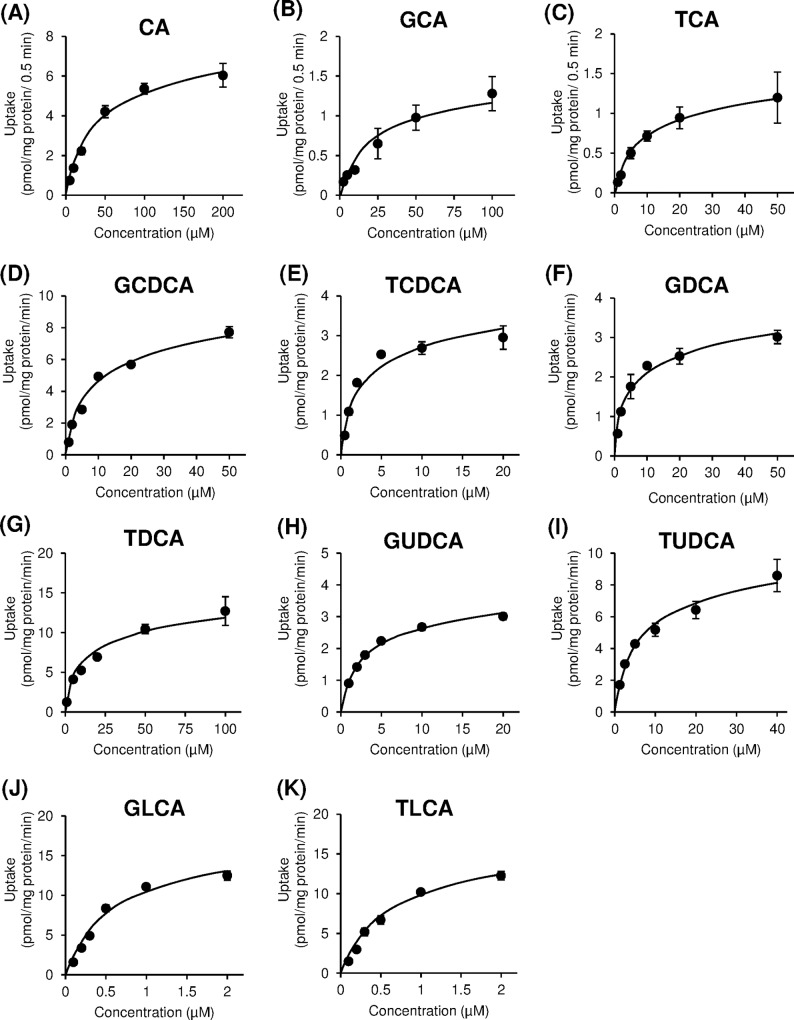
Concentration-dependent uptake of bile acids by organic anion-transporting polypeptide (OATP) 1B1. OATP1B1-overexpressing HEK293 cells were incubated with bile acids at indicated concentrations at 37°C. Each point represents the mean±S.E. (n = 3).

**Fig 4:**
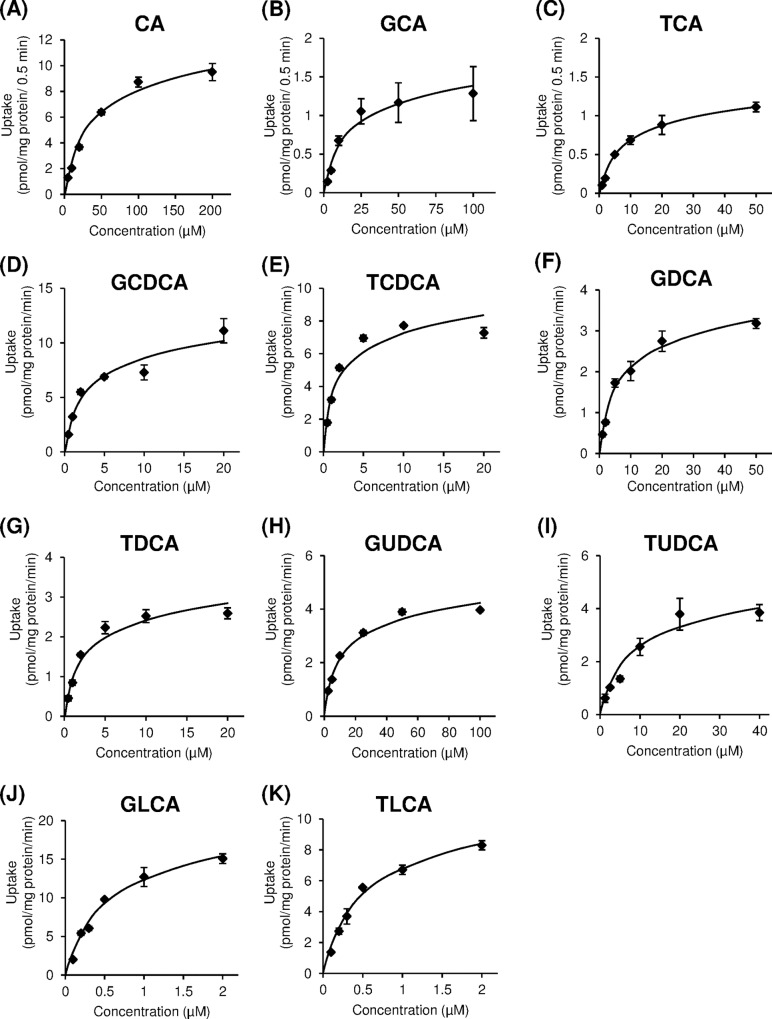
Concentration-dependent uptake of bile acids by organic anion-transporting polypeptide (OATP) 1B3. OATP1B3-overexpressing HEK293 cells were incubated with bile acids at indicated concentrations at 37°C. Each point represents the mean±S.E. (n = 3).

**Fig 5:**
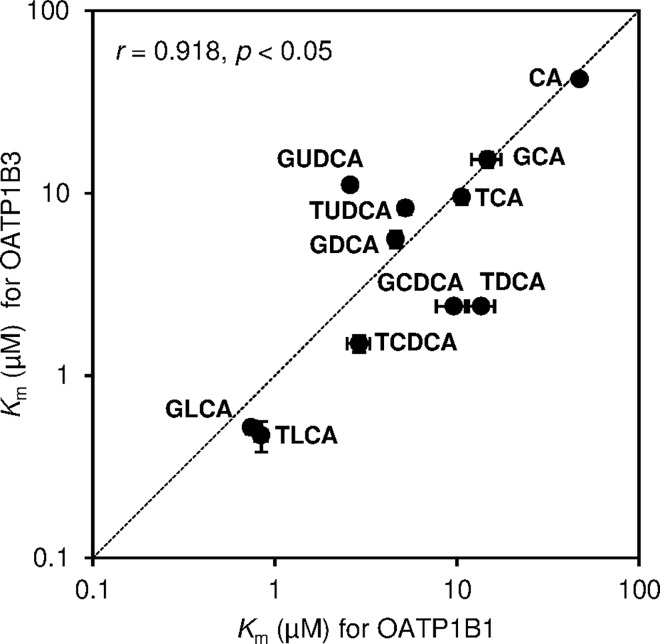
Correlation in *K*_m_ values of bile acids for organic anion-transporting polypeptide (OATP) 1B1 and OATP1B3. *K*_m_ values of bile acids for OATP1B1 are shown on the *X*-axis and those for OATP1B3 are shown on the *Y*-axis. Dotted line in the graph represents 1:1 correlation. Each point represents the mean±S.E. (n = 3).

We examined the initial rate of concentration-dependent transport of bile acids via OATP1B1 and OATP1B3. OATP1B1- and OATP1B3-mediated uptake of bile acids was obtained by subtraction of the uptake by mock cells from the uptake by OATP1B1/HEK293 and OATP1B3/HEK293 cells for each points, respectively. As shown in Figs 3 and 4, both OATP1B1- and OATP1B3-mediated uptake were saturable at higher concentrations of CA and conjugated bile acids, and these uptakes approximated Michaelis-Menten kinetics. The values of kinetic parameters, *K*_m_ and *V*_max_, for bile acids were calculated from Eadie-Hofstee plots, which are summarized in Table 1. The *K*_m_ values of CA for OATP1B1 and OATP1B3 were 47.1 ± 0.6 and 42.2 ± 0.9 μM, respectively, whereas the *K*_m_ values for CDCA and DCA were not determined. The range of *K*_m_ values of conjugated bile acids for OATP1B1 and OATP1B3 were 0.74–14.7 μM and 0.47–15.3 μM, respectively. Moreover, the *K*_m_ values for GLCA (0.74 μM for OATP1B1 and 0.52 μM for OATP1B3) and TLCA (0.84 μM for OATP1B1 and 0.47 μM for OATP1B3) were lower than other bile acids. Therefore, conjugated bile acids were found to exhibit higher affinity as compared to unconjugated bile acid. The *K*_m_ values for OATP1B3 of glycine-conjugated bile acids were 1.1–2.3 times higher than taurine conjugated bile acids. High correlation between *K*_m_ values of bile acids for OATP1B1 and those for OATP1B3 was observed (*r* = 0.918, *p* < 0.05) (Fig 5). The uptake efficiency (*V*_max_/*K*_m_) of GLCA and TLCA via OATP1B1 was 21.5 and 19.6 μL/mg protein/min, and the corresponding *V*_max_/*K*_m_ via OATP1B3 was 29.1 and 21.5 μL/mg protein/min, respectively. The *V*_max_/*K*_m_ of GLCA and TLCA via OATP1B1 and OATP1B3 was 9.8–88 times and 3.5–105 times higher, respectively, than those for other bile acids.

**Table 1:** Kinetic parameters of organic anion-transporting polypeptide (OATP) 1B1- and OATP1B3-mediated uptake of bile acids.

Bile acids	OATP1B1			OATP1B3		
	*K*_m_	*V*_max_	*V*_max_/*K*_m_	*K*_m_	*V*_max_	*V*_max_/*K*_m_
	(μM)	(pmol/mg protein/min)	(μL/mg protein/min)	(μM)	(pmol/mg protein/min)	(μL/mg protein/min)
CA	47.1 ± 0.6	13.3 ± 1.5	0.28	42.2 ± 0.9	21.8 ± 1.7	0.52
GCA	14.7 ± 2.7	3.5 ± 0.9	0.25	15.3 ± 1.5	4.0 ± 0.7	0.27
TCA	10.6 ± 0.3	2.6 ± 0.5	0.24	9.5 ± 0.9	3.2 ± 0.9	0.34
CDCA	+	+	+	+	+	+
GCDCA	9.6 ± 1.9	17.1 ± 9.7	1.6	2.4 ± 0.1	10.3 ± 0.4	4.3
TCDCA	2.9 ± 0.4	4.3 ± 1.0	1.5	1.5 ± 0.2	8.7 ± 0.4	6.1
DCA	+	+	+	+	+	+
GDCA	4.6 ± 0.3	4.9 ± 1.0	1.0	5.6 ± 0.6	3.5 ± 0.1	0.63
TDCA	13.6 ± 2.5	14.8 ± 4.6	1.1	2.4 ± 0.1	2.8 ± 0.2	1.1
UDCA	NT	NT	NT	NT	NT	NT
GUDCA	2.6 ± 0.1	3.7 ± 0.8	1.5	11.1 ± 0.7	5.2 ± 1.7	0.46
TUDCA	5.2 ± 0.4	10.5 ± 2.1	2.0	8.3 ± 0.6	6.6 ± 1.9	0.78
LCA	NT	NT	NT	NT	NT	NT
GLCA	0.74 ± 0.05	15.7 ± 2.1	21.5	0.52 ± 0.04	15.0 ± 2.4	29.1
TLCA	0.84 ± 0.07	16.3 ± 0.8	19.6	0.47 ± 0.09	9.5 ± 0.8	21.5

+: transported but *K*_m_ was not determined, NT: no significant transport was observed. Each data represents the mean ± S.E. (n = 3).

## Discussion

Enterohepatic circulation of bile acids is essential for their own homeostasis. Due to efficient hepatic uptake, bile acids are present at low concentrations in the peripheral circulation. Bile acids are regarded as signaling molecules that play essential roles in many physiological effects. To execute their physiological effects, bile acids need to bind to their specific receptors such as FXR and TGR5 that are expressed not only in liver and intestine but also in tissues outside the enterohepatic circulation. Therefore, the degree of hepatic uptake of the bile acids returning from the intestine may be associated with regulation of bile acid signaling in the whole body. The transporters are key mediators of cellular import and export of bile acids. NTCP and OATPs contribute to the hepatic uptake of bile acids. This study focuses on hepatic uptake of bile acids by OATP1B1 and OATP1B3, which is one of the important processes in their enterohepatic circulation. To investigate the properties of five bile acids (CA, CDCA, DCA, UDCA, LCA) together with their glycine and taurine conjugates transported by OATP1B1 and OATP1B3, individual bile acid uptake experiments were performed using OATP1B1- and OATP1B3-overexpressing HEK293 cells.

In this study, the involvement of transporters in hepatic uptake of CA, CDCA, DCA, UDCA, LCA, and their corresponding glycine and taurine conjugates in OATP1B1- and OATP1B3-overexpressing HEK293 cells was investigated. Our results indicated that glycine and taurine conjugated bile acids and unconjugated bile acids, except for UDCA and LCA, are substrates for both OATP1B1 and OATP1B3 (Fig 2). Previous studies reported that CA, GCA, TCA, GCDCA, TCDCA, TDCA, GUDCA, and TUDCA are substrates of OATP1B1 and OATP1B3 [22–27, 31–35]. However, earlier studies have demonstrated that CA and TCA were not substrates for OATP1B3 [21]. These discrepancies may be explained by differences in the expression level of the transporters in each experimental condition. The present study demonstrated that GCDCA, TCDCA, GDCA, TDCA, GLCA, and TLCA are endogenous substrates of both OATP1B1 and OATP1B3. It has been reported that rat Ntcp accounts for more than 80% of conjugated bile acids (TCA) and less than 50% of unconjugated bile acid (CA) uptake [18, 36, 37]. It has also been reported that the serum concentrations of CA, DCA, and UDCA in Oatp1b2 (high homology to human OATP1B1 and OATP1B3)-null mice are significantly higher as compared to those of wild-type mice [38]. Therefore, Na^+^-independent transport expected to be mainly responsible for the uptake of unconjugated bile acids. However, our results indicated that CDCA and DCA were marginally transported at low concentrations (Fig 2(D) and 2(G)) and UDCA and LCA were not transported by OATP1B1 and OATP1B3 (Fig 2(J) and 2(M)). Similarly, previous studies demonstrated that UDCA was not transported by OATP1B1 and OATP1B3 [27, 39]. Therefore, passive diffusion and/or NTCP and/or other transporters may determine the uptake of UDCA and LCA into hepatocytes in humans.

OATP1B1- and OATP1B3-mediated transport of bile acids was characterized by kinetic analysis. We confirmed that the characteristics of TCA transport (*K*_m_ values of 10.6 ± 0.3 μM and 9.5 ± 0.9 μM for OATP1B1 and OATP1B3, respectively) were similar to those of previous reports [21–23, 31]. The *K*_m_ values for OATP1B1- and OATP1B3-mediated uptake of bile acids in present study and previous reports are summarized in S2 Table. No difference in substrate specificity was observed between OATP1B1 and OATP1B3 in the expression system used for this study.

In this study, we characterized glycine and taurine conjugated bile acids and CA transport by OATP1B1 and OATP1B3. The rank order of kinetic parameters (*K*_m_, *V*_max_ and *V*_max_/*K*_m_) of CA and its glycine and taurine conjugates via OATP1B1 and OATP1B3 was CA > GCA ≥ TCA (Table 1). The difference between conjugated and unconjugated bile acids is the presence of a C-24 conjugation. Conjugation with glycine or taurine reduces p*K*a of bile acids, improves water solubility, and reduces lipophilicity [40]. Previous reports state that p*K*a values of unconjugated bile acids are 5–6, while those of glycine and taurine conjugated bile acids are 4–5 and 1–2, respectively [18]. Therefore, under conditions (pH 7.4), unconjugated bile acids can cross cell membranes by passive nonionic diffusion because approximately 10% of unconjugated bile acids are present as nonionic type, while conjugated bile acids (glycine and taurine) require transporter mediated uptake. However, no significant correlation was observed between apparent 1-octanol/water partition coefficient (logP_ow_) of bile acids (S3 Table) and *K*_m_ values for OATP1B1 and OATP1B3 (*r* = −0.279, *p* = 0.406 and *r* = −0.369, *p* = 0.264, respectively). Similarly, no significant correlation was observed between distribution coefficient (logD_oct_) of bile acids and *K*_m_ values for OATP1B1 and OATP1B3 (*r* = 0.146, *p* = 0.668 and *r* = 0.0748, *p* = 0.827, respectively). It is suggested that transport properties of bile acids may be associated with chemical structure rather than lipophilicity.

The *K*_m_ values for CDCA and DCA were not determined in the present study. Previous report suggested that OATP1B1 and OATP1B3 play important roles in CDCA uptake into the liver using 7-nitrobenz-2-oxa-1,3-diazole (NBD)-labeled CDCA [41]. The *K*_m_ values for OATP1B1- and OATP1B3- mediated CDCA-NBD uptake were 1.45 ± 0.39 μM and 0.54 ± 0.09 μM, respectively. These results indicated that C-24 chemical modification of bile acids might change the transport properties. Further investigations on the transport of bile acids by using OATP1B1 and OATP1B3 mutants and evaluation of chemical modification of the amino acid residues of OATP1B1 and OATP1B3 may provide us with detailed information.

Moreover, the rank order of *V*_max_/*K*_m_ of the conjugated bile acids for OATP1B1 and OATP1B3 was found to be conjugated LCA > conjugated CDCA; conjugated DCA and conjugated UDCA ≥ conjugated CA (Table 1). This difference of *V*_max_/*K*_m_ among the bile acids may result from variation in the chemical properties of their steroidal hydroxylation pattern (rank order of *V*_max_/*K*_m_: monohydroxy bile acids > dihydroxy bile acids ≥ trihydroxy bile acids). Bile acids are potentially cytotoxic at high concentrations and exhibit pathological effects such as plasma membrane damage, mitochondrial oxidative stress, and endoplasmic reticulum-mediated apoptosis [42]. The cytotoxicity of bile acids is considered to be associated with their degree of hydrophobicity, which depends on the number of hydroxylation sites (monohydroxy bile acids > dihydroxy bile acids > trihydroxy bile acids) [43]. In liver, bile acids can undergo sulfation and glucuronidation as well as conjugation with glycine and taurine for detoxification and elimination from the body. Therefore, hepatic uptake of bile acids by OATP1B1 and OATP1B3 may play an important role in the first step of detoxification of cytotoxic bile acids.

We assessed the variability of *K*_m_ value of bile acid transport for OATP1B1, OATP1B3, and NTCP. The *K*_m_ values of bile acids, except GDCA and TDCA, were slightly lower and wider in range for OATP1B1 and OATP1B3 than NTCP (S1 Fig) [44]. The *K*_m_ values of GLCA and TLCA for OATP1B1 and OATP1B3 were approximately 7–14 fold lower than *K*_m_ values of GLCA and TLCA for NTCP. These results indicated that the affinity of bile acids for OATP1B1 and OATP1B3 was higher than that for NTCP. Previous studies reported the presence of hepatic lobular concentration gradient for the uptake of bile-acid analog [45]. It suggested that periportal hepatocytes are more active than centrolobular cells in sequestering bile acids. Because rat Ntcp was evenly distributed across the liver lobe [46], it is expected to be similar in human NTCP. In contrast to OATP1B1, which is expressed in hepatocytes throughout the liver lobe, OATP1B3 is highly expressed around the central vein [23, 47, 48]. Whether this specific expression is related to a specific physiological function remains unknown. However, the present study suggests that most of the conjugated bile acids are transported not only by NTCP but also by OATP1B1 into periportal hepatocytes. The secondary bile acids and unconjugated bile acids, which are relatively in high concentration, may be transported into hepatocytes around the central vein by OATP1B3. Further investigations are required to determine the contribution of Na^+^-dependent and Na^+^-independent transport mechanisms and other transporters (i.e., NTCP, OATP1B1, and OATP1B3) to the uptake of individual bile acids using human hepatocytes.

In conclusion, we showed that GCDCA, TCDCA, GDCA, TDCA, GLCA, and TLCA are novel endogenous substrates of both OATP1B1 and OATP1B3. Our results suggest that glycine and taurine conjugated bile acids were preferred to unconjugated bile acids as substrates for OATP1B1 and OATP1B3. It is further suggested that OATP1B1 and OATP1B3, as well as NTCP, may play important roles in the hepatic uptake of bile acids.

## Supporting Information

S1 Fig Comparison of *K*_m_ values of bile acid transport for organic anion-transporting polypeptide (OATP) 1B1, OATP1B3, and Na+-taurocholic acid cotransporting polypeptide (NTCP). (A) *K*_m_ values of bile acids for OATP1B1 are shown on the *X*-axis, and those for NTCP cited from the previous report [44] are shown on the *Y*-axis; (B) *K*_m_ values of bile acids for OATP1B3 are shown on the *X*-axis, and those for NTCP are shown on the *Y*-axis. Dotted line in the graphs represents 1:1 correlation. Each point represents the mean ± S.E. (n = 3)
(TIF)

S1 Table Analysis of selected reaction monitoring (SRM) parameters and internal standards for bile acids. Bile acids and internal standards were detected under negative electrospray ion mode [M-H]− in the SRM.
(PDF)

S2 Table *K*_m_ values of organic anion-transporting polypeptide (OATP) 1B1- and OATP1B3-mediated uptake of bile acids. (PDF)

S3 Table Experimentally determined apparent 1-octanol/water partition coefficient (P_ow_) of bile acids. The 1-octanol/water partition coefficients of bile acids were measures based on Organisation for Economic Co-operation and Development (OECD) guidelines for testing of chemicals: Partition Coefficient (n-octanol/water): Shake Flask Method (OECD 107, 1995). Bile acids (10 μM) were dissolved in 3–6 mL of 1-octanol presaturated with KH buffer (adjusted to pH 7.4) in a 10-mL glass tube. About 3–6 mL of KH buffer (adjusted to pH 7.4) presaturated with 1-octanol was added and the glass tube was shaken by hand approximately hundred times for 5 minutes, and centrifuged at 3000 rpm for 30 min at 24°C. The resulting two phases were carefully separated and bile acid concentration in both the phases was measured using LC/MS/MS. The calculating formula for logD_oct_ is as follows, logD_oct_ = logP_ow_—log(1 + 10^pH^-^pKa^). The p*K*a values of unconjugated, glycine conjugated, and taurine conjugated bile acids are 5, 4, and 1, respectively.
(PDF)
