# Supplementary figures and images for: Preference of Conjugated Bile Acids over Unconjugated Bile Acids as Substrates for OATP1B1 and OATP1B3
Source: PLoS One. 2017 Jan 6;12(1):e0169719. doi: 10.1371/journal.pone.0169719 (PMC5218478; doi:10.1371/journal.pone.0169719)

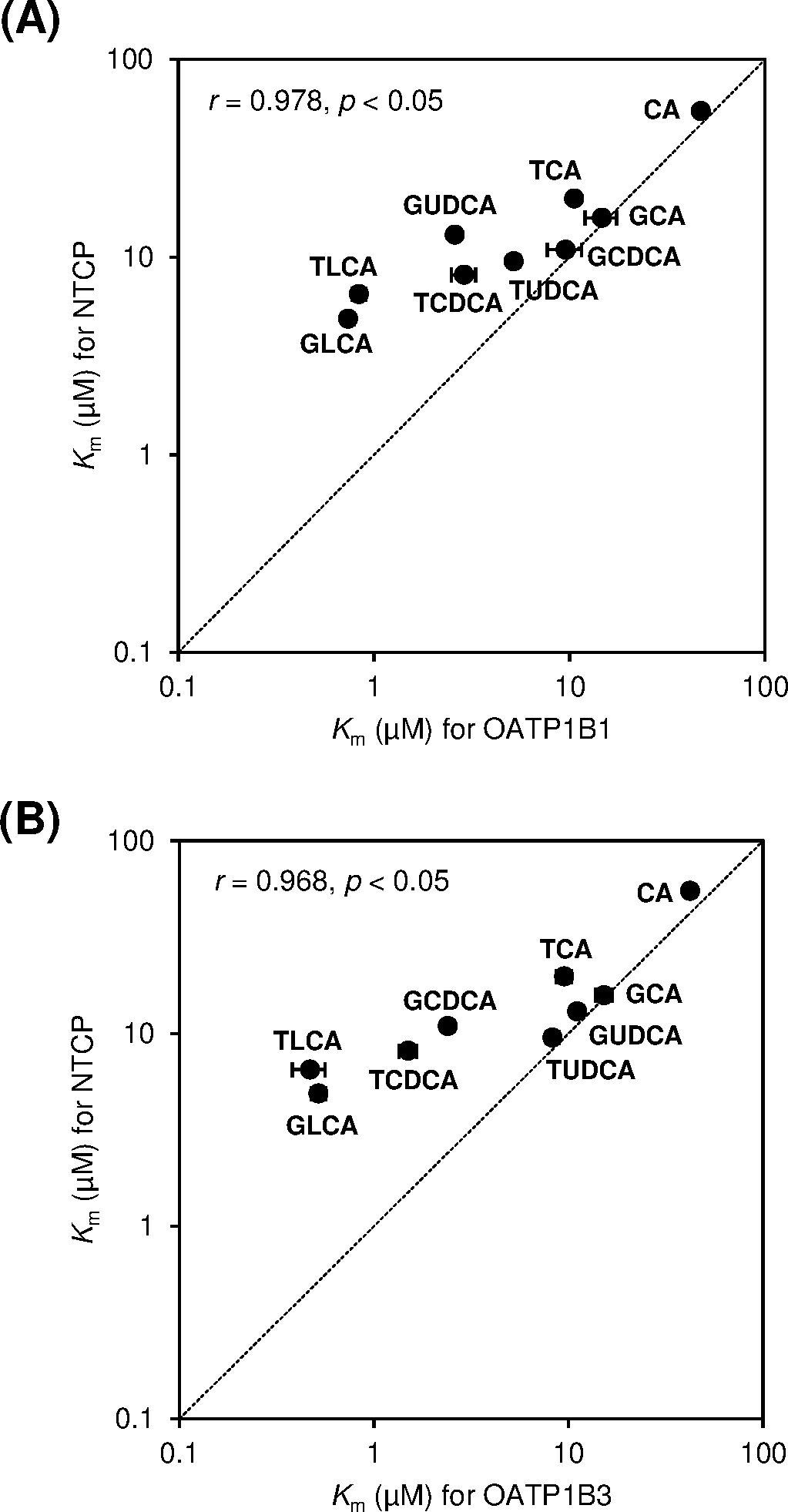

Supplement: S1 Fig — (A) Km values of bile acids for OATP1B1 are shown on the X-axis, and those for NTCP cited from the previous report [44] are shown on the Y-axis; (B) Km values of bile acids for OATP1B3 are shown on the X-axis, and those for NTCP are shown on the Y-axis. Dotted line in the graphs represents 1:1 correlation. Each point represents the mean ± S.E. (n = 3) (TIF) [file pone.0169719.s001.tif]
