# Supplementary material for: Preference of Conjugated Bile Acids over Unconjugated Bile Acids as Substrates for OATP1B1 and OATP1B3
Source: PLoS One. 2017 Jan 6;12(1):e0169719. doi: 10.1371/journal.pone.0169719 (PMC5218478; doi:10.1371/journal.pone.0169719)
Supplement: S1 Table — Bile acids and internal standards were detected under negative electrospray ion mode [M-H]− in the SRM. (PDF) [file pone.0169719.s002.pdf]

S1 Table. Analysis of selected reaction monitoring (SRM) parameters and internal standards for bile acids.

Analytes	SRM transition	Fragmentor voltage (V)	Collision Energy (eV)	Internal standard
CA	*m/z* 407 → 407	230	0	3, 7, 12-[^{18}O]CA
GCA	*m/z* 464 → 74	210	45	3, 7-[^{18}O]GCA
TCA	*m/z* 514 → 80	300	95	3, 7-[^{18}O]GCA
CDCA	*m/z* 391 → 391	210	0	3, 7-[^{18}O, $^{2}H_2$]GCDCA
GCDCA	*m/z* 448 → 74	200	40	3, 7-[^{18}O, $^{2}H_2$]GCDCA
TCDCA	*m/z* 498 → 80	290	90	3, 12-[^{18}O, $^{2}H_2$]TDCA
DCA	*m/z* 391 → 391	200	0	3-[^{18}O, $^{2}H_2$]GLCA
GDCA	*m/z* 448 → 74	200	40	3, 7-[^{18}O, $^{2}H_2$]GCDCA
TDCA	*m/z* 498 → 80	290	75	3, 12-[^{18}O, $^{2}H_2$]TDCA
UDCA	*m/z* 391 → 391	230	0	3, 7, 12-[^{18}O]CA
GUDCA	*m/z* 448 → 74	200	40	3, 7-[^{18}O]GCA
TUDCA	*m/z* 498 → 80	290	75	3, 7-[^{18}O]GCA
LCA	*m/z* 375 → 375	190	0	3-[^{18}O, $^{2}H_2$]GLCA
GLCA	*m/z* 432 → 74	190	40	3-[^{18}O, $^{2}H_2$]GLCA
TLCA	*m/z* 482 → 80	280	75	3-[^{18}O, $^{2}H_2$]TLCA
3, 7, 12-[^{18}O]CA	*m/z* 413 → 413	230	0	
3, 7-[^{18}O]GCA	*m/z* 470 → 74	210	45	
3, 7-[^{18}O, $^{2}H_2$]GCDCA	*m/z* 454 → 74	200	40	
3, 12-[^{18}O, $^{2}H_2$]TDCA	*m/z* 504 → 80	290	75	
3-[^{18}O, $^{2}H_2$]GLCA	*m/z* 435 → 74	190	40	
3-[^{18}O, $^{2}H_2$]TLCA	*m/z* 485 → 80	280	75	
