# Supplementary material for: Preference of Conjugated Bile Acids over Unconjugated Bile Acids as Substrates for OATP1B1 and OATP1B3
Source: PLoS One. 2017 Jan 6;12(1):e0169719. doi: 10.1371/journal.pone.0169719 (PMC5218478; doi:10.1371/journal.pone.0169719)
Supplement: S2 Table — (PDF) [file pone.0169719.s003.pdf]

S2 Table. K_m values of organic anion-transporting polypeptide (OATP) 1B1- and OATP1B3-mediated uptake of bile acids.

Bile acids	Expression system	OATP1B1	OATP1B3	Reference
		K_m (μM)	K_m (μM)	
CA	HEK293	47.1 ± 0.6	42.2 ± 0.9	Present study
	HEK293	11.4		[21]
	Oocyte		41.8	[24]
GCA	HEK293	14.7 ± 2.7	15.3 ± 1.5	Present study
	Oocyte	ND	ND	[34]
	Oocyte		43.4	[24]
	Trophoblast	ND	ND	[35]
TCA	HEK293	10.6 ± 0.3	9.5 ± 0.9	Present study
	Oocyte	13.6		[22]
	HEK293	33.8		[25]
	HEK293	ND	ND	[33]
	HEK293	10.0		[21]
	Oocyte	ND	ND	[34]
	Oocyte		5.8 ± 1.2	[23]
	MDCKII		112	[26]
	Oocyte		42.2	[24]
	HEK293	21.3		[32]
	HEK293	8.52		[31]
CDCA	HEK293	+	+	Present study
GCDCA	HEK293	9.6 ± 1.9	2.4 ± 0.1	Present study
	Trophoblast	ND	ND	[35]
TCDCA	HEK293	2.9 ± 0.4	1.5 ± 0.2	Present study
	Oocyte	ND	ND	[24]
DCA	HEK293	+	+	Present study
GDCA	HEK293	4.6 ± 0.3	5.6 ± 0.6	Present study
TDCA	HEK293	13.6 ± 2.5	2.4 ± 0.1	Present study
	Oocyte	ND	ND	[24]
UDCA	HEK293	NT	NT	Present study
GUDCA	HEK293	2.6 ± 0.1	11.1 ± 0.7	Present study
	HEK293	5.17	24.7	[27]
TUDCA	HEK293	5.2 ± 0.4	8.3 ± 0.6	Present study
	HEK293	7.47	15.9	[27]
LCA	HEK293	NT	NT	Present study
GLCA	HEK293	0.74 ± 0.05	0.52 ± 0.04	Present study
TLCA	HEK293	0.84 ± 0.07	0.47 ± 0.09	Present study

ND: not determined, +: transported but K_m was not determined, NT: no significant transport was observed.

Each data represents the mean ± S.E. (n = 3).
