# Supplementary material for: Preference of Conjugated Bile Acids over Unconjugated Bile Acids as Substrates for OATP1B1 and OATP1B3
Source: PLoS One. 2017 Jan 6;12(1):e0169719. doi: 10.1371/journal.pone.0169719 (PMC5218478; doi:10.1371/journal.pone.0169719)
Supplement: S3 Table — The 1-octanol/water partition coefficients of bile acids were measures based on Organisation for Economic Co-operation and Development (OECD) guidelines for testing of chemicals: Partition Coefficient (n-octanol/water): Shake Flask Method (OECD 107, 1995). Bile acids (10 μM) were dissolved in 3–6 mL of 1-octanol presaturated with KH buffer (adjusted to pH 7.4) in a 10-mL glass tube. About 3–6 mL of KH buffer (adjusted to pH 7.4) presaturated with 1-octanol was added and the glass tube was shaken by hand approximately hundred times for 5 minutes, and centrifuged at 3000 rpm for 30 min at 24°C. The resulting two phases were carefully separated and bile acid concentration in both the phases was measured using LC/MS/MS. The calculating formula for logDoct is as follows, logDoct = logPow—log(1 + 10pH-pKa). The pKa values of unconjugated, glycine conjugated, and taurine conjugated bile acids are 5, 4, and 1, respectively. (PDF) [file pone.0169719.s004.pdf]

S3 Table. Experimentally determined apparent 1-octanol/water partition coefficient (P_{ow}) of bile acids.

Bile acids	P_{ow}	$logP_{ow}$	$logD_{oct}$
CA	2.4 ± 0.3	0.38	−2.0
GCA	0.09 ± 0.01	−1.02	−4.4
TCA	0.40 ± 0.04	−0.40	−6.8
CDCA	264 ± 27	2.42	0.018
GCDCA	3.6 ± 0.2	0.56	−2.8
TCDCA	1.8 ± 0.1	0.25	−6.2
DCA	351 ± 16	2.55	0.15
GDCA	6.1 ± 0.3	0.78	−2.6
TDCA	3.8 ± 0.2	0.58	−5.8
UDCA	73 ± 3	1.87	−0.53
GUDCA	0.59 ± 0.03	−0.23	−3.6
TUDCA	0.62 ± 0.07	−0.21	−6.6
LCA	28335 ± 6546	4.44	2.0
GLCA	554 ± 17	2.74	−0.66
TLCA	469 ± 17	2.67	−3.7

Each data represents the mean ± S.D. (n = 6).
